# Animal Movement and Establishment of Vaccinia Virus Cantagalo Strain in Amazon Biome, Brazil

**DOI:** 10.3201/eid1704.101581

**Published:** 2011-04

**Authors:** Jociane Cristina Quixabeira-Santos, Maria Luiza G. Medaglia, Caroline A. Pescador, Clarissa R. Damaso

**Affiliations:** Author affiliations: Universidade Federal do Rio de Janeiro, Rio de Janeiro, Brazil (J. Quixabeira-Santos, M.L.G. Medaglia, C.R. Damaso);; Universidade Federal do Mato Grosso, Cuiabá, Brazil (J. Quixabeira-Santos, C.A. Pescador);; Instituto de Defesa Agropecuária do Estado de Mato Grosso, Cuiabá (J. Quixabeira-Santos)

**Keywords:** Viruses, zoonoses, vaccinia virus, Cantagalo virus, poxvirus, animal movement, virus spread, Brazil, dispatch

## Abstract

To understand the emergence of vaccinia virus Cantagalo strain in the Amazon biome of Brazil, during 2008–2010 we conducted a molecular and epidemiologic survey of poxvirus outbreaks. Data indicate that animal movement was the major cause of virus dissemination within Rondônia State, leading to the establishment and spread of this pathogen.

Outbreaks of vaccinia virus (VACV) infection in dairy cows and dairy workers have been frequently reported in Brazil during the past decade, mainly within the southeastern region (*1**–**6*), except for central Goiás (*2*) and Tocantins State in the northeastern boundary of the Brazilian Amazon biome (Figure 1) (*7*). VACV Cantagalo strain (CTGV) was first detected in 1999 and associated with several outbreaks (*1**,**5**,**7*). Related strains have been reported in subsequent outbreaks (*2**–**4**,**6*).

**Figure 1:**
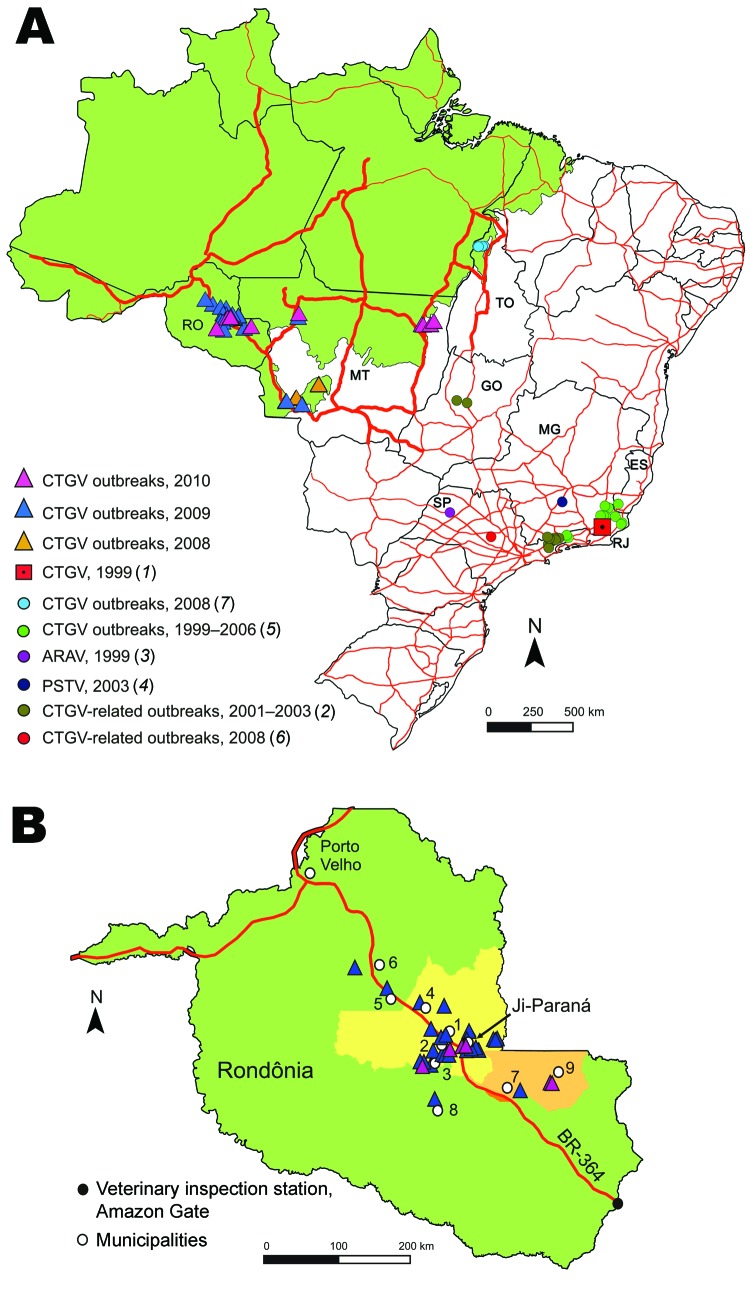
Location of vaccinia virus Cantagalo strain (CTGV)–related outbreaks. A) Brazilian states where CTGV-related outbreaks have been reported. RJ, Rio de Janeiro; SP, São Paulo; ES, Espírito Santo; MG, Minas Gerais; GO, Goiás; TO, Tocantins; MT, Mato Grosso; RO, Rondônia. B) An enlarged map of Rondônia showing the location of the outbreaks along highway BR-364. The 2 largest dairy regions and the municipalities referred in this article are also shown: 1, Ouro Preto D’Oeste; 2, Teixeirópolis; 3, Urupá; 4, Jaru; 5, Cacaulândia; 6, Ariquemes; 7, Cacoal; 8, Nova Brasilândia D’Oeste; 9, Espigão D’Oeste. Green shading, Amazon biome; light yellow shading, main dairy region; light orange shading, second-largest dairy region. Thin red lines indicate the Brazilian highway network and thick red lines the main highways of the Amazon region. Maps were constructed by using ArcGIS version 9.3.1 (www.esri.com) based on the location coordinates. ARAV, vaccinia virus Araçatuba strain; PSTV, vaccinia virus Passatempo strain.

Mato Grosso State is partially inserted into the southeastern Amazon biome (Figure 1) and has the largest cattle population of Brazil, ≈26 million (*8*). It is the main connection with the Amazon states, especially with Rondônia. Rondônia has nearly 11.2 million cattle and is the largest milk producer in the region, yielding >720 million liters/year (*8*). Therefore, intense livestock trade occurs through the so-called Amazon Gate of Mato Grosso and Rondônia. Both states had previously been considered poxvirus disease–free.

## The Study

From June 2008 through June 2010, we investigated outbreaks of poxvirus-related disease on 56 dairy farms of Mato Grosso and Rondônia (Figure 1). A probable case was defined as illness observed in any cattle from these farms that showed clinical signs consistent with a poxvirus-related infection: vesicopustular lesions on the udder and/or teats of cows or on muzzle and/or tongue of suckling calves, fever, lymphadenopathy, and remission after ≈3 weeks. A confirmed case was any probable case that was laboratory confirmed by PCR or that had an epidemiologic link to a farm with PCR-positive results. We considered as epidemiologic links the migration of dairy workers, animal movements, and location of neighboring farms. Each inspection generated an epidemiologic investigation form by the animal health agencies of these states. Human infection was noted with a description of lesions on hands, arms, and face.

A total of 52 samples of scabs were collected from cattle on 23 farms. DNA was isolated as described (*5*) and subjected to PCR by using the EACP primers to detect the full-length hemagglutinin (HA) gene, a marker for orthopoxvirus infection (*1**,**5**,**7*). To specifically detect CTGV, we used a reverse primer that annealed to the flanking regions of an 18-nt deletion in the HA gene, a molecular signature of the CTGV-like strains from Brazil (*1**,**3**,**5*). DNA from reference CTGV (*1*) was used as positive control, and VACV-WR and cowpox virus strain Brighton Red were used as negative controls (*5*). This strategy successfully identified CTGV-like isolates, as confirmed by DNA sequencing (*5**,**7*). Thirty-five samples, representative of the 23 farms (Table 1), were positive for both orthopoxvirus (full-length HA) and CTGV infections. This result strongly suggests that CTGV was the etiologic agent of disease in these farms. For further confirmation, we sequenced the HA, K2L, and C7L genes from 6 samples, as described (*7*). All sequences showed the 18-nt deletion in the HA gene as well as the 15-nt deletion within K2L found in all CTGV-like isolates (*7**,**9*). The relatedness of the virus isolates with CTGV-like isolates was confirmed by analysis of the nucleotide identity matrix (Table 2) and phylogenetic inference (data not shown).

**Table 1:** Results of testing PCR analysis and investigation of sick animals during outbreaks of vaccinia virus Cantagalo strain infection in the Amazon biome, Brazil, 2008–2010

Farm locations	No. farms tested/total no. farms*	No. sick animals/total no. animals										Cohort†
Age <12 mo			Age 13–23 mo			Age >24 mo			Total
F	M		F	M		F	M		
Mato Grosso	12/21	26/535	28/491		0/579	0/412		277/1,611	0/510		331/4,138	331/2,637 (12.55)
Rondônia	11/35	117/1,287	95/1,823		0/1006	0/892		688/4,660	0/893		900/10,561	900/7,770 (11.58)
Total	23/56	143/1,822	123/2,314		0/1,585	0/1,304		965/6,271	0/1,403		1,231/14,699	1,231/10,407 (11.83)

*Of the 56 affected farms, 23 had samples collected for PCR analysis. All farms tested had positive results for vaccinia virus Cantagalo strain. †No. sick animals/total no. animals (%). Cohort consisted of female cattle >24 mo of age (considered lactating cows) and all calves <12 mo of age.

**Table 2:** Nucleotide identity scores obtained for the concatenated alignment of HA, C7L, and K2L genes of Mato Grosso and Rondônia isolates and distinct vaccinia virus strains, Brazil, 2008–2010*

Strain	VACV-Cop	VACV-Lister	VACV-WR	ARAV	CTGV- MU-07	CTGV	CTGV-VSD-01	CTGV-URU-04	CTGV-URU-06	CTGV-JP-11	CTGV-JP-13	CTGV-ESP-01
VACV-Cop	ID	0.990	0.990	0.971	0.971	0.971	0.971	0.971	0.971	0.971	0.971	0.971
VACV-Lister	0.990	ID	0.991	0.972	0.972	0.972	0.972	0.972	0.972	0.972	0.972	0.972
VACV-WR	0.990	0.991	ID	0.976	0.976	0.976	0.976	0.976	0.976	0.976	0.976	0.976
ARAV	0.971	0.972	0.976	ID	0.999	0.999	0.999	0.999	0.999	0.999	0.999	0.999
CTGV-MU-07	0.971	0.972	0.976	0.999	ID	0.999	0.999	0.999	0.999	0.999	0.999	0.999
CTGV	0.971	0.972	0.976	0.999	0.999	ID	0.999	0.999	0.999	0.999	0.999	0.999
CTGV-VSD-01	0.971	0.972	0.976	0.999	0.999	0.999	ID	1.000	1.000	1.000	1.000	1.000
CTGV-URU-04	0.971	0.972	0.976	0.999	0.999	0.999	1.000	ID	1.000	1.000	1.000	1.000
CTGV-URU-06	0.971	0.972	0.976	0.999	0.999	0.999	1.000	1.000	ID	1.000	1.000	1.000
CTGV-JP-11	0.971	0.972	0.976	0.999	0.999	0.999	1.000	1.000	1.000	ID	1.000	1.000
CTGV-JP-13	0.971	0.972	0.976	0.999	0.999	0.999	1.000	1.000	1.000	1.000	ID	1.000
CTGV-ESP-01	0.971	0.972	0.976	0.999	0.999	0.999	1.000	1.000	1.000	1.000	1.000	ID

*GenBank accession nos. for vaccinia virus genomes are as follows: VACV-Cop (Copenhagen strain), M35027; VACV-Lister, AY678276; VACV-WR (Western Reserve strain), NC_006998. Individual GenBank accession nos. for HA, C7L and K2L genes of CTGV and CTGV-related viruses are as follows, respectively: ARAV (Araçatuba virus) (*3*): AY523994, EF051277, EF175987); CTGV isolate MU- 07 (*7*): FJ545689, FJ545688, FJ545687; CTGV (reference sample) (*1*): AF229247, EF488959, EU528619; CTGV isolates investigated in this work VSD-01(municipality of Vale de São Domingos, MT, 2008): HQ336388, HQ336394, HQ336400; URU-04 (municipality of Urupá, RO, 2009): HQ336387, HQ336393, HQ336399; URU-06 (municipality of Urupá, RO, 2009): HQ336389, HQ336395, HQ336401; JP-11 (municipality of Ji-Paraná, RO, 2010): HQ336385, HQ336391, HQ336397; JP-13 (municipality of Ji-Paraná, RO, 2010): HQ336384, HQ336390, HQ336396; ESP-01 (municipality of Espigão D’Oeste, RO, 2010): HQ336386, HQ336392, HQ336398. HA, hemagglutinin; VACV, vaccinia virus; CTGV, vaccinia virus CantaIago strain; MT, Mato Grosso State; RO, Rondônia State; ID, identical viruses.

An epidemiologic investigation was conducted by using data available from the 56 epidemiologic investigation forms, analyzing cattle movement forms issued during this period, and interviewing farmers and animal health agency veterinarians. In addition to the 23 farms with positive samples, 33 other farms had epidemiologic links to CTGV-positive farms. Therefore, our data suggest that CTGV infection was the cause of all outbreaks.

Analysis showed disease prevalence among lactating cows and suckling calves to be 11.83% (1,231/10,407) (Table 1). These rates were lower than those for farms of southeastern states, which reached 50% to 80% (*10*). Factors that may account for this difference were more efficient surveillance systems in Mato Grosso and Rondônia and the notably larger herd size on Mato Grosso and Rondônia farms. In this study, 55.36% of the affected farms had >100 cattle (mean=253), in contrast to 90% of southeastern farms, which had <100 cattle (*5**,**10*). The economic losses were substantial because milk production was the major activity at 76.79% of the farms in this study; the income of 93.02% of those was solely based on dairy activities. In addition, 67.85% of the farms reported at least 1 infected dairy worker.

**Figure 2:**
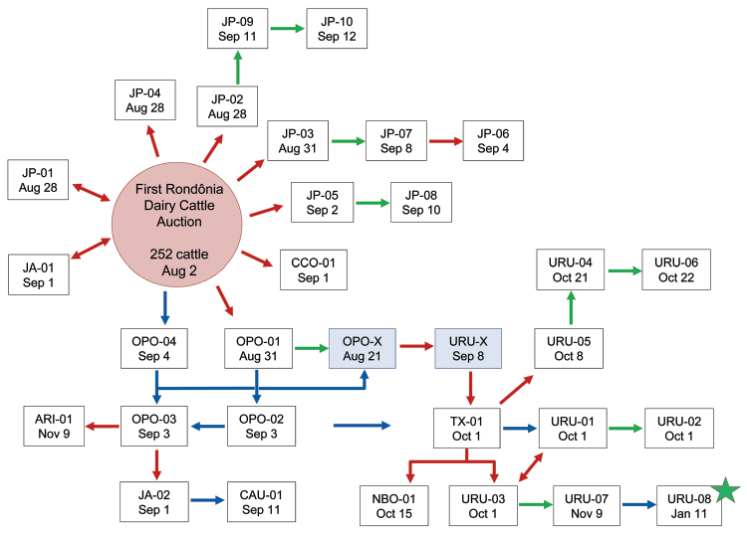
Spread of vaccinia virus Cantagalo (CTGV) strain infection and the epidemiologic links between affected farms in Rondônia, 2009. Two farms provided 252 cattle for the First Rondônia Dairy Cattle Auction (10°51′02.38′′S, 61°59′23.93′′W), which occurred in Ji-Paraná, Rondônia, on August 2, 2009. Eighty-one animals were sold to 6 farms; the remaining cattle were returned to their original owners. Dairy workers were hired as temporary workers for the pre-event period. Each farm affected by CTGV infection in 2009 is represented by a white box with date of disease notification to the Rondônia animal health agency indicated. The report usually happened 2–15 days after onset of clinical signs, but occasionally veterinarians were contacted after lesions had healed. Light blue boxes indicate farms whose owners did not report sick animals but which traded cattle with CTGV-affected farms or had close contact with them; the date of animal trading is shown. Epidemiologic links between farms are indicated by arrow colors (red for animal trade, blue for migration of workers, green for neighboring farms) but do not infer geographic distances. Farms were located in these municipalities: JP, Ji-Paraná; JA, Jaru; CCO, Cacoal; OPO, Ouro Preto D’Oeste; ARI, Ariquemes; CAU, Cacaulândia; TX, Teixeirópolis; NBO, Nova Brasilândia D’Oeste; URU, Urupá. Green star indicates a farm where animals showed clinical signs December 13, 2009, but the owner did not notify the state agency until January 11, 2010.

BR-364 is a 4,141.5-km highway with a major veterinary inspection station, the Amazon Gate, at the Mato Grosso–Rondônia border. Affected farms were mostly located along this main road connecting Mato Grosso with the Amazon region (Figure 1, panel A). Despite that, we could not establish an epidemiologic link between the outbreaks in Mato Grosso and Rondônia. In contrast, the spread and establishment of CTGV within Rondônia were evidently related to animal movement along BR-364. Outbreaks in Rondônia (Figure 1, panel B) were first reported in August 2009 after the First Rondônia Dairy Cattle Auction in the municipality of Ji-Paraná (Figures 1, 2), which is part of Rondônia’s major dairy region (*11*). According to auction records, animals were not traded from other states for this event. Therefore, the circumstances of CTGV entry into Rondônia are still uncertain, but informal animal trade should be considered as well as the pre-event translocation of temporary dairy workers from Mato Grosso, as reported by some farmers.

Cattle trade during the auction launched virus spread throughout the state, which was amplified by subsequent trading and human translocation (Figure 2). Animal movement was associated with the disease spread on 48.57% of farms, whereas migrating dairy workers and neighboring farms were associated with 22.86% and 28.57% of the cases, respectively. Conversely, in Mato Grosso, 13.04% of the farms were related to the virus spread by animal movement, whereas 60.87% were associated with migrating workers. On southeastern farms, dairy workers have also been associated with virus spread, but no quantitative data have been reported (*5**,**10*). The distinct pattern in Rondônia could be related to extraordinary intrastate cattle movement. Analysis of 459,884 cattle movement forms issued in 2009 revealed an intrastate translocation of 6,875,031 cattle, which corresponded to 29.34 cattle/km^2^ in contrast to 17.44 cattle/km^2^ in Mato Grosso. The occurrence of new CTGV outbreaks in 2010 supports a nonsporadic pattern of infection in Rondônia, highlighting the establishment of this zoonotic infection within the state.

## Conclusions

This study investigated the appearance, establishment, and spread of CTGV infection into the Amazon biome. Our findings suggest that animal movement was the main cause of virus long-distance dissemination in Rondônia, whereas the migration of dairy workers was involved in the focal spread. Animal movement plays an essential role in cross-border spread of several disorders worldwide, including bovine tuberculosis, foot-and-mouth disease, and bovine diarrhea viruses (*12**–**15*). We suggest animal movement is also a notable risk factor for CTGV dissemination. According to the inspection station database at the Amazon Gate, 287,906 cattle were moved into Rondônia through BR-364 in 2008–2009, of which 12,501 stayed in Rondônia. Attention is needed to address the westward spread of this zoonosis into the Amazon biome, toward the third largest dairy region of Rondônia surrounding Porto Velho (Figure 1, panel B). Therefore, new regulations and intensified surveillance should be implemented to restrain CTGV spread, particularly in dairy regions with elevated rates of cattle trade for heifer replacements.
